# Notch signalling drives bone marrow stromal cell-mediated chemoresistance in acute myeloid leukemia

**DOI:** 10.18632/oncotarget.7964

**Published:** 2016-03-07

**Authors:** Paul Takam Kamga, Giulio Bassi, Adriana Cassaro, Martina Midolo, Mariano Di Trapani, Alessandro Gatti, Roberta Carusone, Federica Resci, Omar Perbellini, Michele Gottardi, Massimiliano Bonifacio, Armel Hervé Nwabo Kamdje, Achille Ambrosetti, Mauro Krampera

**Affiliations:** ^1^ Stem Cell Research Laboratory, Section of Hematology, Department of Medicine, University of Verona, Verona, Italy; ^2^ Division of Hematology, Ca' Foncello Hospital, Treviso, Italy; ^3^ Department of Biomedical Sciences, University of Ngaoundere-Cameroon, Cameroon

**Keywords:** AML, MSC, Notch, chemoresistance

## Abstract

Both preclinical and clinical investigations suggest that Notch signalling is critical for the development of many cancers and for their response to chemotherapy. We previously showed that Notch inhibition abrogates stromal-induced chemoresistance in lymphoid neoplasms. However, the role of Notch in acute myeloid leukemia (AML) and its contribution to the crosstalk between leukemia cells and bone marrow stromal cells remain controversial. Thus, we evaluated the role of the Notch pathway in the proliferation, survival and chemoresistance of AML cells in co-culture with bone marrow mesenchymal stromal cells expanded from both healthy donors (hBM-MSCs) and AML patients (hBM-MSCs*). As compared to hBM-MSCs, hBM-MSCs* showed higher level of Notch1, Jagged1 as well as the main Notch target gene HES1. Notably, hBM-MSCs* induced expression and activation of Notch signalling in AML cells, supporting AML proliferation and being more efficientin inducing AML chemoresistance than hBM-MSCs*. Pharmacological inhibition of Notch using combinations of Notch receptor-blocking antibodies or gamma-secretase inhibitors (GSIs), in presence of chemotherapeutic agents, significant lowered the supportive effect of hBM-MSCs and hBM-MSCs* towards AML cells, by activating apoptotic cascade and reducing protein level of STAT3, AKT and NF-κB.

These results suggest that Notch signalling inhibition, by overcoming the stromal-mediated promotion of chemoresistance,may represent a potential therapeutic targetnot only for lymphoid neoplasms, but also for AML.

## INTRODUCTION

Acute myeloid leukemia (AML) is the most common malignant myeloid disorder in adulthood, characterized by the blockage of myeloid differentiation and blast cell accumulation in the bone marrow [1]. One of the major challenges in AML, with the exception of promyelocytic acute leukemia, is still related to the high relapse rate after chemotherapy due to the persistence of residual blast cells in the bone marrow. In this phenomenon, stromal cell components of bone marrow niche, deriving from human mesenchymal stromal cells (hBM-MSCs), play a crucial role by supporting leukemia cell survival and growth, thus leading to escape from chemotherapy-induced apoptosis [2-5]. The crosstalk between leukemia cells and stromal microenvironment is complex and involves different pathways, including Wnt/β-catenin, Sonic Hedgehog and Notch. Notch is a developmental and evolutionary pathway that controls proliferation, self-renewal, differentiation and survival of normal and cancer cells. Notch signalling consists of four receptors, Notch1 to Notch4, and five ligands including Jagged1, Jagged2, Dll1, Dll3 and Dll4. The binding of the ligand to its cognate receptor induces the release of the Notch Intracellular Cleaved Domain (NICD) that, in turn,migrates into the nucleus. Then, in association with co-activators, such as C-promoter Binding Factor 1 (CBF1) and Mastermind-like proteins (MAML), NICD trans-activates target genes, including Hairy and Enhancer of Split related gene-1 (*HES-1*). Depending on the cell system, Notch signalling may act as tumor suppressor or oncogene [6]. In hematological malignancies, activating mutations of Notch1 are found in 50% of T-cell acute lymphoblastic leukemia (T-ALL) [7, 8]. Similarly, chronic lymphocytic leukemia (CLL) and B-cell acute lymphoblastic leukaemia cells (B-ALL) express Notch receptors and ligands and are sensitive to Notch signalling inhibitors [4, 5, 9, 10].

In myeloid counterpart the role of Notch remains controversial: some authors described Notch expression and activation in AML samples and AML cell lines, but the pathway was slightly activated, as demonstrated by the low expression level of Notch target genes [11-13]. Consistently, they demonstrated that the reactivation of Notch signalling induced apoptosis and differentiation of blast cells into mature cells [11, 12]. By contrast, the activation of Notch pathway in AML has been associated to bad prognosis [14]. Moreover, patients with hyper-expression of Notch1 displayed poorer overall survival [15]. Recently, Grieselhuber and co-worker identified Notch expression and activation in acute promyelocytic leukemia, demonstrating that both genetic and pharmacologic inhibition of Notch signalling abrogated the enhanced self-renewal of blast cells [16]. However, the effect of exogenous micro-environmental Notch signalling on AML cell survival and response to chemotherapy has not yet been evaluated.

In the present study, we demonstrate that hBM-MSCs isolated from patients with AML (hBM-MSCs*) possess a different Notch expression pattern as compared to hBM-MSCs obtained from healthy donors. Moreover, hBM-MSCs* display more pronounced chemo-protective features. Using pharmacological inhibition of Notch pathway or specific blocking antibodies, we found that Notch blockade inhibits AML proliferation and abrogates hBM-MSC-mediated AML resistance to chemotherapeutic agents through the modulation of pro-/anti-apoptotic proteins balance and levels of prosurvival proteins, including AKT, STAT3 and NF-kB. Overall, these findings suggest for the first time that Notch signalling is important for chemo-resistance in the bone marrow microenvironment of AML patients, thus representing a potential target to improve AML treatment.

## RESULTS

### Notch signalling is expressed by hBM-MSCs from AML patients

**Figure 1:**
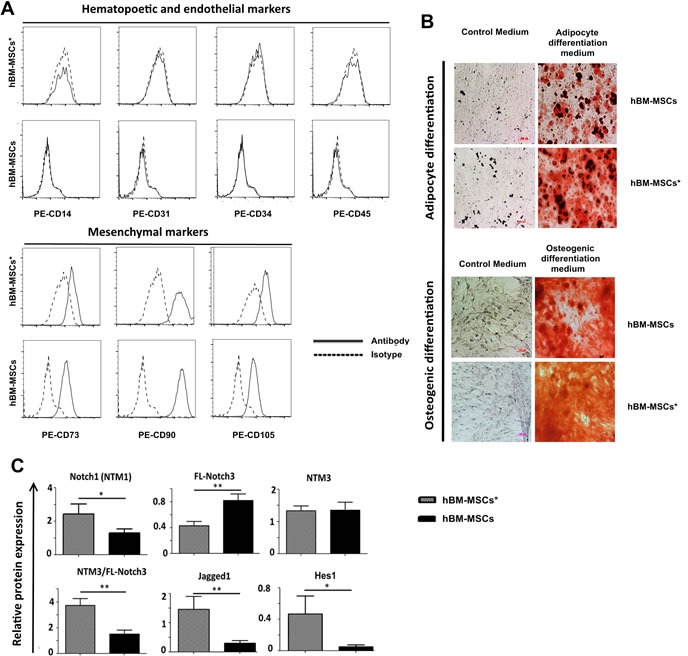
Notch expression in bone marrow MSCs isolated from healthy donors (hBM-MSCs) and AML patients (hBM-MSC*) **A.** Flow cytometry analysis of MSCs for expression of hematopoetic, endothelial and mesenchymal markers. **B.** Oil Red O staining (upper panel) and Alizarin Red staining (lower panel) of mesenchymal cells grown during 21 days in adypocyte and osteogenic differentiation media, respectively. **C.** Relative quantification of protein expression after densitometry analysis of immunoblots. Protein expression levels were normalized to expression levels of GAPDH, data are expressed as the mean ± SEM of 12 patients and 12 healthy donors *p < 0.05, **p < 0.01. HEK-293 cell line was used as positive control. NTM: Notch Trans-Membrane domain; FL: Full Length; EC: Notch Extracellular Cleaved domain. As indicated in the datasheet, anti-Notch4 detected 3 different isoforms (a, b and c).

To address a possible role for Notch signalling in the interaction between stromal microenvironment and leukemia cells, we first examined the expression of Notch pathway component in hBM-MSC from 12 healthy donors (hBM-MSC) and 12 AML patients (hBM-MSC*). hBM-MSCs and hBM-MSC* were isolated through plastic adherence and characterized for mesenchymal markers expression (Figure 1A) and multilineage differentiation capacities (Figure 1B). Notch expression by hBM-MSCs and hBM-MSCs* was quantified through Western blot followed by densitometry analysis, by using Image J-software. Notch receptors are generally expressed in different forms including the Full-Length receptor (FL, 200-280 kDa) and the Notch Trans-Membrane form (NTM, 90-110kDa). We found that hBM-MSCs and hBM-MSCs* expressed all the four Notch receptors as well as the Notch ligand Jagged1 (Figure S1). Importantly, the mean level of Notch1 and Jagged1 proteins was higher in hBM-MSCs* than in hBM-MSCs (Figure 1C). By contrast, Notch3 was less represented in hBM-MSCs* as compared to hBM-MSCs, while the two cell types displayed the same level of NTM3. Accordingly, NTM3/FL-Notch3 ratio was higher in hBM-MSCs* than in hBM-MSCs. Differently from hBM-MSCs, which expressed low to undetectable levels of the Notch target gene Hes1, hBM-MSCs*showed higher level of Hes1 protein, thus suggesting that active Notch signalling is operational in bone marrow microenvironment of AML patients (Figure 1C).

### hBM-MSCs modulate Notch expression in AML cells, supporting survival of primary AML cells

**Figure 2:**
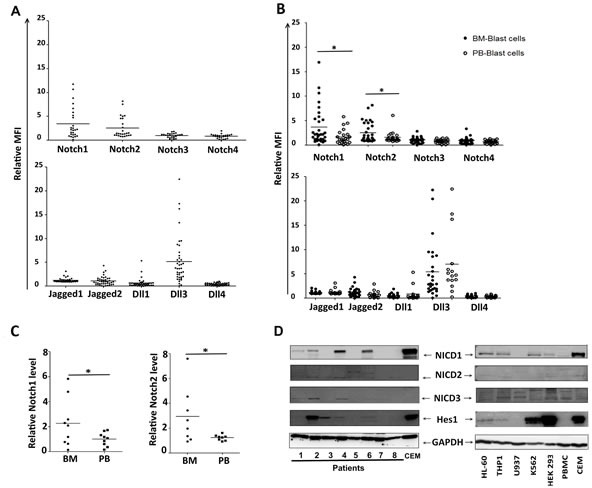
Notch expression and activation in AML cells **A.** FACS analysis of AML cells (*n* = 43) using fluorochrome-conjugated antibodies specific for extracellular Notch receptors and ligands. **B.** A comparison of the expression level of each component was carried out between leukemia cells from peripheral blood (PB) and leukemia cells from bone marrow (BM) **C.** In a subset of 9 patients, Notch1 and Notch2 levels were quantified in PB and BM from the same patient, and Mann-Whitney test was used to analyze the differences between means (*p < 0.05). In A, B and C, data were represented as relative Mean of Fluorescence Intensity (MFI). **C.** Representative western blots analysis for Hes1 and activated form of Notch receptors (NICD1, NICD2, NICD3) in AML samples (left) and in cell lines (right). Data are representative of 4 independent experiments; HEK-293 and CEM cell lines were used as positive controls.

Overexpression and activation of Notch signalling in hBM-MSCs* from AML patients suggested a specific Notch signalling involvement in the bone marrow niche for the crosstalk between AML cells and stromal cells. A first attempt to validate this hypothesis was to analyse the expression of Notch components in AML cells isolated from peripheral blood (PB, *n* = 16) and from bone marrow (BM, *n* = 28). Globally, through FACS analysis (Figure S1C), we found a significant expression of Notch components in all the samples, with high levels of Notch1, Notch2, Jagged2 and Dll3 (Figure 2A). Regardless of the FAB and cytogenetic subtype, all BM samples showed higher levels of Notch1 and Notch2 as compared to PB samples (Figure 2B). To further validate this finding, we confirmed the higher levels of Notch1 and Notch2 expression in BM as compared to PB samples in a subset of 9 patients in which both BM and PB samples were available at diagnosis (Figure 2C). Noteworthy, the presence of Notch receptors on cell surface did not correlate with the signalling activation status. Indeed, only a subset of patients showed active Notch system, as revealed by the presence of Hes1, NICD1, NICD2 and NICD3 (Figure 2D). Similarly, Western blot analysis showed the presence of NICD1, NICD2, NICD3 and Hes1 in some AML cell lines, namely HL-60 and THP1 (Figure 2C, right). Notably, the expression of all these molecules was affected by the treatment with GSI (Figures S2A, S2B). In all the AML cell lines we also confirmed the presence, at variable levels, of Notch1 and Notch3 receptors, Jagged1, Jagged2 (only in THP1 cell line), Dll1, Dll3 and Dll4 ligands (data not shown). Overall, the presence of the active form of the receptors suggested that Notch activation was related to the three receptors, leading to multiple regulation levels of Notch activation, including compensation, synergism and antagonism.

**Figure 3:**
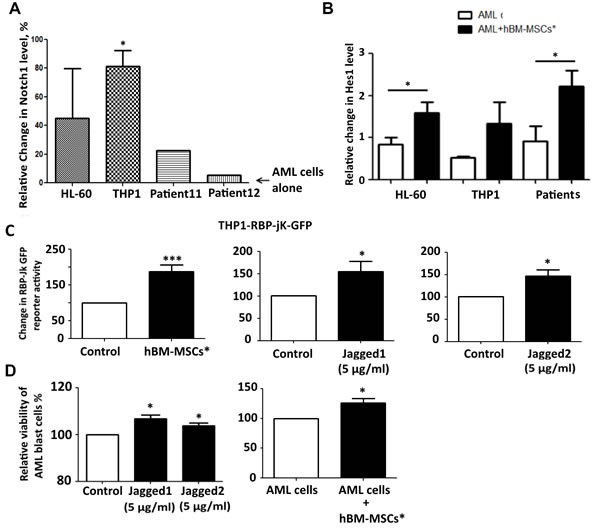
Modulation of Notch expression and activation in AML and hBM-MSCs* upon co-culture **A.** Representative change in Notch1 level in AML cells: after 24 hours of co-culture with hBM-MSCs*, AML cells were collected and flow cytometry analysis for Notch1 expression was performed. The relative MFI was calculated and normalized as percentage with relative MFI of AML cells cultured alone. Data are expressed as mean ± SEM of 4 independent experiments. **B.** Representative western blot analysis of Hes1 in AML cells after 24 hours of co-culture with hBM-MSCs*. **C.** Representative RBP-jk activity in THP1 cells expressing RBP-jk-GFP reporter gene, THP1 cells were transfected with RBP-jk GFP reporter gene, after 4 hours cells were treated with Notch ligands or seeded on hBM-MSCs* monolayer. GFP levels were analyzed through flow cytometry after 72hours of co-culture. Data were normalized in percentage with THP1 transfected with CMV-GFP plasmid and are expressed as mean ± SEM of 4 independent experiments. **D.** Viability of primary leukemia cells cultured in presence of Notch ligands (Jagged1, Jagged2) or co-cultured with hBM-MSCs*. After 4 days, cells were collected and stained with Topro-3 to discriminate death cells through flow cytometry analysis. Data are representative of mean ± SEM of 3-7 independent experiments involving 3-10 patients.

To establish whether the interaction between stromal cells and AML cells involves Notch pathway, we co-cultured AML cells with hBM-MSCs*. After 24 hours, we performed the immunophenotyping of Notch receptors and ligands on AML cells, thus finding the increase of Notch1 level (Figure 3A). To assess whether this change in expression was correlated to Notch pathway activation, we investigated the change in the Notch target gene expression in AML cell lines upon co-culture with hBM-MSCs*. Co-cultured AML cells showed the increase of Hes1 level as well as NICD1 (Figures 3B, S2B), which was abrogated after medium supplementation with GSIs (Figure S2B). Consistently, THP1 cells transfected with RBP-Jk GFP reporter and seeded on hBM-MSCs* showed enhanced GFP signal when normalized with THP1 transfected with CMV-GFP plasmid (Figure 3C), and the increase in RBP-Jk GFP activity was similar to that observed when cells were challenged with Notch receptors ligands (Figure 3C). Importantly, hBM-MSCs* as well as Notch ligands were capable to promote the survival of AML primary cells in co-culture or in culture, respectively, as shown by the reduction of Topro-3 positive cells after 4 days of culture (Figure 3D). These data indicate that the Notch pathway may represent a mechanism through which stromal microenvironment and leukemia cells reciprocally interact inside the bone marrow niche, eventually promoting AML cell survival.

### Notch inhibition suppresses AML proliferation

**Figure 4:**
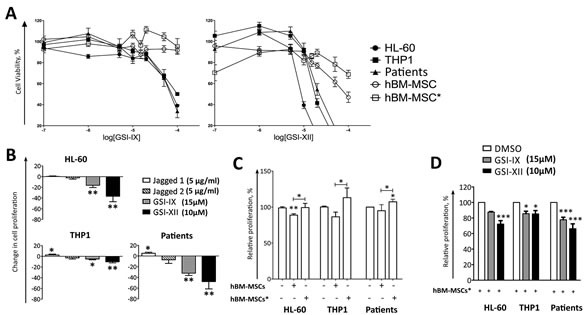
Notch signalling controls AML cell proliferation in culture and in co-culture with hBM-MSCs* **A.** AML cells were cultured with increasing concentrations of GSI-IX or GSI-XII for 48 hours,then cell viabilitywas analyzed through MTT assay. **B.** In order to quantify cell proliferation, CFSE-stained AML cells were cultured for 4 days in the presence of Notch modulators. Data are normalized by comparing each condition with untreated or DMSO treated wells. **C.** Effects of hBM-MSC and hBM-MSC* on the proliferation of AML cells in absence of Notch modulators. Data are compared with the proliferation of AML cultured alone or with DMSO that was normalized to 100% of cell proliferation. **D.** Effects of GSIs on AML cell proliferation in co-culture with hBM-MSCs*. Data are compared with AML/hBM-MSCs* culture condition, which was normalized to 100% of cell proliferation. Data are expressed as the mean ± SEM of 5-10 independent experiments involving 10 patients: *p < 0.05, **p < 0.01, **p < 0.001.

Previous studies showed that artificial activation of Notch signalling in AML cells, through NICD over-expression, decreased cell viability by inducing apoptosis [11, 12]; conversely, activation with recombinant ligands produces contrasting effects [13, 17, 18]. Here, we observed that only leukemia cells expressing Hes1 and at least one Notch receptor cleaved/active form, as expected, were sensitive to *in vitro* treatment with GSIs (Figure S3A). We found that viability of the cell lines (THP1, HL-60) and primary AML cells displaying active Notch signalling (Figure 2D) was reduced in a dose-dependent manner by using GSIs, as measured by MTT assay; by contrast, hBM-MSCs and hBM-MSCs* viability was not affected by GSIs, except for higher concentration (Figure 4A). However, we observed that the main effect of GSIs and SAHM1, a pharmacological inhibitor of MAML1, was related to the inhibition of cell proliferation more than induction of cell death (Table S3). To further confirm these observations, AML cells were stained with CFSE before GSI treatment (15μM for GSI-IX and 10μM for GSI-XII) to evaluate cell proliferation. GSI-treated AML cells showed high CFSE staining after 4 days of culture, as compared to DMSO (vehicle of GSIs)-treated cells, corresponding to GSI-induced inhibition of AML cell proliferation (Figure 4B). Consistently, the presence of Jagged1 slightly increased the proliferation of THP1 and primary leukemia cells. To confirm the specificity of Notch modulators, each of them was used to treat THP1 transfected with RBP-Jk-GFP reporter plasmid. As above mantioned, the addition of ligands increased GFP signal, while inhibitors had the opposite effect (Figures 3C, S2C).

We then investigated the effect of stromal cells on AML cell proliferation. We found that hBM-MSCs inhibited AML cell proliferation, while hBM-MSCs* supported the proliferation of THP1 and primary leukemia cells (Figure 4C). The addition of GSIs to co-culture reverted the supportive effect of hBM-MSCs* and increased the suppressive properties of hBM-MSCs towards AML cell proliferation (Figure 4D). Likewise, pan-inhibition of Notch using the Notch transcription factor inhibitor SAHM1, reduced AML cell proliferation without any effect on cell death (Figure S3 and Table S3).

### AML cell lines silenced for RBP-jk are more sensible to chemotherapy

**Figure 5:**
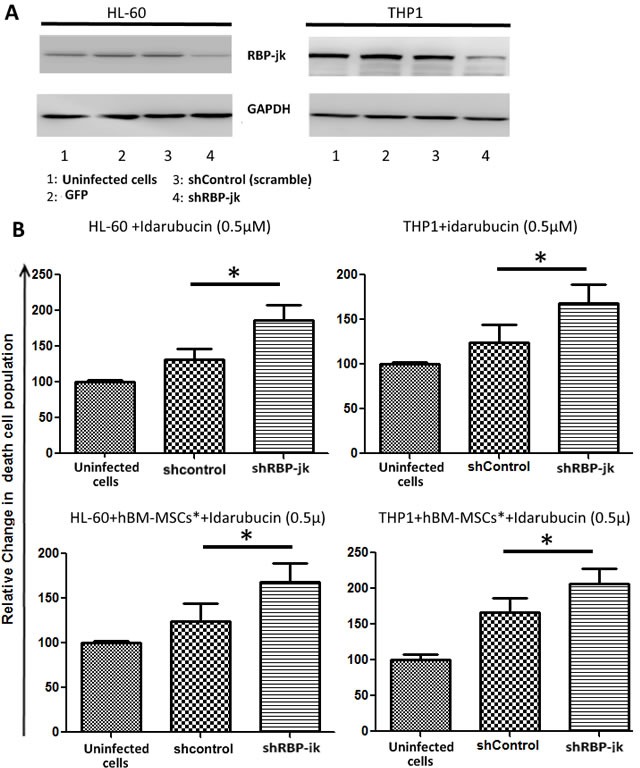
Drug sensibility of AML cell lines with RBP-jK knock down **A.** Notch transcription factor regulator RBP-jk was silenced in AML cell lines HL-60 and THP1 using lentiviral particles expressing shRNA directed to RBP-jk. Knock down cells were treated with Idarubucin, in presence or absence of hBM-MSCs*. After 48 hours of treatment, AML cells were harvested and stained with PI to analyse cell viability. Data are expressed as mean ± SEM of three independent experiments:* *p* < 0.05

As hBM-MSC-mediated Notch signalling was involved in survival and proliferation of AML cells, we hypothesized that this contribution could be extended to AML response to chemotherapy. To validate this hypothesis, we decided to take advantage of a loss of function model of AML cells by silencing Notch in cells with active Notch pathways. As recombination signal-binding protein Jκ (RBP-jk) is a key transcription factor downstream of receptor activation in Notch signalling pathway, its down-regulation by using short interfering RNA is largely used to mimic pan-Notch inhibition in different cancer cells systems (19,20). We therefore proceed to knocked-down RBP-jk in two AML cell lines displaying active Notch signalling, namely HL-60 and THP1 (Figure 2D). Infection of these cell lines with shRBP-jk effectively inhibited RBP-jk in HL-60 and THP1 (Figure 5A). We found that silenced cells were more sensible to treatment with Idarubucine than their counterpart infected with a non-specific shRNA (Figure 5B). This observation suggested that activation of Notch in AML by stromal microenvironment promotes chemoresistance.

### Notch/GSI-inhibition abrogates drug resistance induced by stromal cells

**Figure 6:**
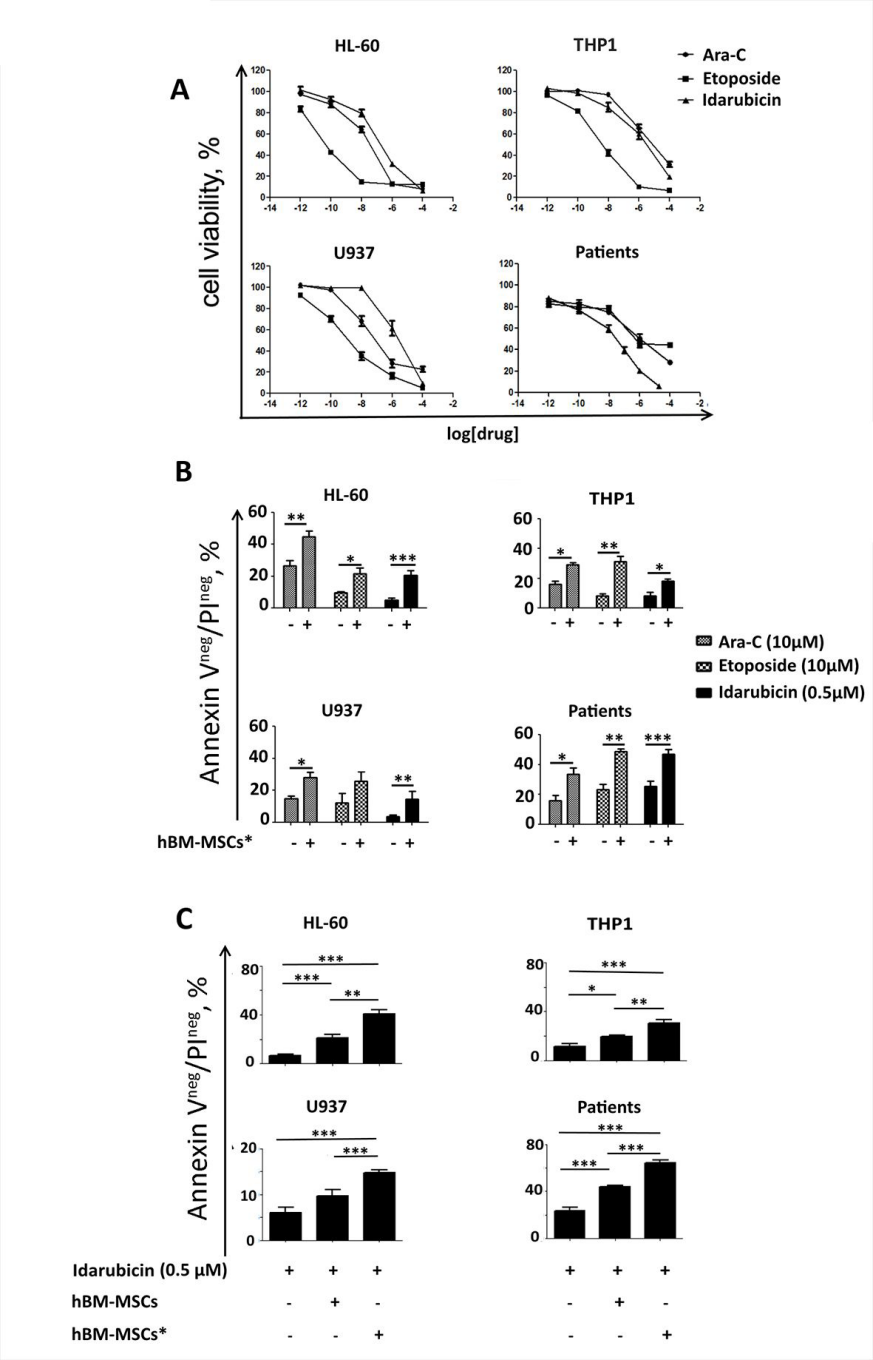
hBM-MSCs* promote AML chemo-resistance **A.** AML cells were cultured with increasing concentrations of chemotherapeutic agents (Ara-C, Etoposide and Idarubicine). After 48 hours, cell viability was analyzed with MTT assay to identify the EC50. Graphs are representative of 4 independent experiments. **B.** Cell lines and primary AML cells were treated with Ara-C, Etoposide or Idarubicin in presence or absence of hBM-MSCs*, and then stained with AnnexinV/PI in order to evaluate cell apoptosis. **C.** Cell lines and primary AML cells were treated with Idarubicin in presence or absence of hBM-MSCs or hBM-MSCs* and then stained with AnnexinV/PI and analysed through flow cytometry. Data are expressed as the mean ± SEM of 5-10 independent experiments involving 10 patients: *p < 0.05, **p < 0.01, **p < 0.001

We and other groups have previously demonstrated that bone marrow-derived stromal cells could protect leukemia cells from chemotherapy-induced apoptosis (2,4,5,21). To understand whether hBM-MSCs* could promote chemoresistance, we treated AML cells with drugs routinely used for AML therapy, i.e. Ara-C, Idarubicin and Etoposide. After 48 hours of treatment, cell viability was analyzed by MTT assay. As expected, drug treatment reduced AML cell survival in a dose-dependent manner (Figure 6A). We next confirmed that stromal cells support AML cell survival by preventing drug-induced apoptosis (Figure 6B). Notably, hBM-MSCs* displayed a stronger protective activity towards AML cells in presence of Idarubicin, as compared to hBM-MSCs (Figure 6C).

**Figure 7:**
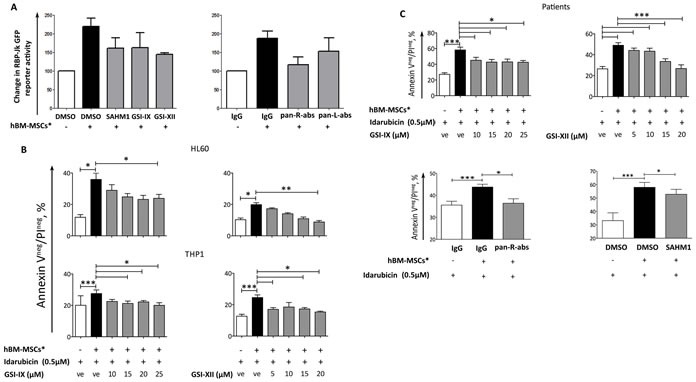
Notch inhibitors abrogate hBM-MSC*-induced chemoresistance **A.** THP1 cells were transfected with RBP-Jk GFP reporter gene; prior to transfection, cells were treated with Notch modulators, which were maintained in the medium during and after transfection. After 4 hours from transfection, cells were seeded on hBM-MSCs* monolayer. GFP levels were analyzed by flow cytometry after 72 hours of co-culture. Data were normalized in percentage with THP1 transfected with CMV-GFP plasmid and are expressed as mean ± SEM of 2 independent experiments. **B.** AML cell lines were cultured alone or co-cultured with hBM-MSCs* in presence of Idarubicin (0.5 μM) and with increasing concentrations of GSIs. **C.** Primary AML cells were cultured alone or co-cultured with hBM-MSCs* in presence of Idarubicin (0.5 μM) and either increasing concentrations of GSIs or Notch transcription factor inhibitor SAHM1 (20μM) or a combination of anti-Notch1, 2, 3 and 4 blocking antibodies (pan-R-abs, pan Receptor blocking antibodies). All blocking antibodies were used at a final concentration of 5μg/ml. After 48 hours of treatment, AML cells were harvested and stained with AnnexinV/PI to analyze cell viability. Data are represented as mean ± SEM of 3 independent experiments. One way ANOVA was used for statistical analysis: * *p* < 0.05, **p < 0.01, ***p < 0.001. “ve”: vehicle = DMSO.

As GSI-IX, GSI-XII, SAHM1 and blocking antibodies were capable of reverting hBM-MSCs*-dependent Notch activation in AML cells (Figure 7A), we therefore analyzed the viability, upon Notch inhibition, of drug-treated AML cells in co-culture with hBM-MSCs*. We found that GSIs treatment increased the chemosensitivity of both cell lines and primary AML towards Idarubicin treatment (Figure 7B, 7C and Table S4). The same effect was obtained when leukemia cells were treated with Ara-C or Etoposide (Table S4) and in cell contact-independent setting by using Transwell^®^ system (Figure S4). Although GSIs are good Notch inhibitors, they still have secondary targets, such as proteasome (22), thus displaying potential toxicity that may limit their clinical application. Thus, we confirmed that GSI and Idarubicin treatment did not affect hBM-MSCs* viability and morphology (Figures S5A and S5B). Alternatively, the use of blocking antibodies is a current strategy for the treatment of many types of cancers [19]. Hence, we tested whether anti-Notch blocking antibodies were capable of abrogating hBM-MSC*-mediated chemoresistance of AML cells. The addition of a mixture of anti-Notch1, anti-Notch2, anti-Notch3 and anti-Notch4 (pan-R-abs) or specific Anti-Notch blocking antibodies alone, significantly enhanced drug sensitivity of AML cells in co-culture with hBM-MSC* (Figures 7C, S6). The addition of SAHM1 reverted only partially the chemoresistance, thus suggesting a role for MAML1-independent Notch signalling activation in the stroma and AML crosstalk (Figure 7C).

### Notch inhibition is associated with the activation of apoptotic cascade and decrease of prosurvival proteins, such asAKT, STAT3and NF-κB

**Figure 8:**
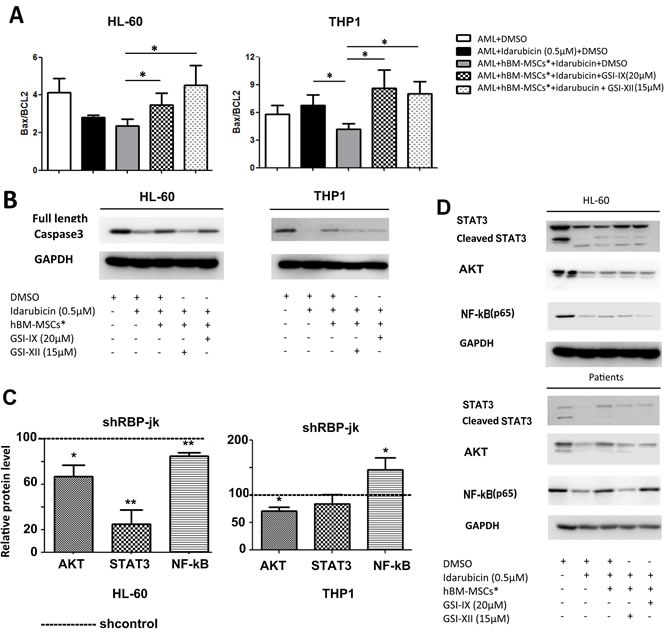
Notch inhibition controls Bax/Bcl-2 ratio and protein levels of Akt, NF-kB and Stat3 AML cells were cultured alone or co-cultured with hBM-MSCs* in presence of Idarubicin (0.5 μM) and either GSI-IX (20μM) or GSI-XII(15μM). **A.** After 24 hours of incubation, cells were collected washed, fixed, permeabilized, stained with anti-Bax-Alexa Fluor 488 and anti-Bcl-2-Alexa Fluor 488, and then analyzed through flow cytometry. Data are expressed as mean ± SEM of 3 independent experiments: *p < 0.05, **p < 0.01, ***p < 0.001. **B.** Western blot analysis of Caspase3. **C.** Relative levels of AKT, NF-kB and STAT3 in shRBP-jk cells, cells in culture were washed, fixed, permeabilized and probed with corresponding primary antibody, followed by staining with PE-conjugated secondary antibody. Cells were then analysed through flow cytometry. Data are expressed as mean ± SEM of 3 independent experiment experiments *p < 0.05, **p < 0.01. **D.** Western blot analysis of Caspase3 AKT, STAT3, NF-κB. Data are representative of 3-4 patients.

It has been previously shown that bone marrow stromal cells prevent apoptosis of AML cells through upregulation of anti-apoptotic proteins [21, 24]. We then assessed whether Notch inhibition influenced Bcl-2 family proteins. We found that hBM-MSCs* co-culture led to the decrease of pro-apototic Bax / anti-apoptotic Bcl-2 ratio (Figure 8A) and the level of full-length caspase3 expression (Figure 8B). By contrast, GSIs were capable of increasing this ratio and reducing the level of full-length caspase3. These observations suggested that Notch inhibition could revert hBM-MSCs-mediated AML chemoresistance by modulating the balance between pro-apoptotic and anti-apoptotic of the Bcl-2 family protein and the level of full-length caspase3. In parallel, we observed that cell lines with lower level of RBP-jk displayed reduced levels of AKT, STAT3 and NF-kB expression (Figure 8C). Interestingly, these proteins have been linked to poor prognosis and represent good targets to overcome drug resistance [20-22]. We therefore investigated, upon pharmacological inhibition of Notch, the activation and expression levels of AKT, STAT3 and NF-kB proteins. Activation status was screened through Western blot analysis of phospho-AKT (Thr308), phospho-STAT3(Tyr705), and phospho-NF-κB (Ser536) expression. However, the pattern of phosphorylation was not homogeneous among cell lines and AML cells, thus revealing a variable degree of activation of these pathways in hBM-MSC*-mediated AML chemoresistance (data not shown). By contrast, Idarubicin treatment reduced the level of NF-κB, STAT3 and AKT expression in AML cells after 24 hours of treatment. This decrease was partially abrogated when AML cells were co-cultured on hBM-MSCs*, and the presence of GSIs in co-culture medium restored Idarubicin-dependent reduction of AKT, NF-κB and STAT3 proteins (Figure 8D).

## DISCUSSION

Many of the most recent therapeutic approaches have shifted from targeting cancer cells to interfering with the microenvironment, as the influence on tumor cells of tissue microenvironment has been clearly shown to play a pivotal role in cancer cell chemoresistance and treatment failure [23]. In hematological malignancies, the presence of stromal cells promotes leukemic cell escape from apoptosis induced by chemotherapy(2,5,21), and leukemia development is often associated to persistent abnormalities in the microenvironment fostering neoplastic cell growth [24]. Amongst them, Wnt/β-catenin, Sonic Hedgehog and Notch pathways play an important role by regulating the crosstalk between leukemia cells and stromal microenvironment [25]. For instance, some activating mutations of β-catenin in osteoblast compartment are sufficient to generate AML-like disease in mouse model [26]. Interestingly, the accumulation of nuclear β-catenin induces Jagged1 expression in stromal cells, and the genetic or pharmacological inhibition of Notch signalling ameliorates the symptoms in mice, thus indicating the pathogenic role of Notch in AML [26]. In line with these observations, we demonstrate here that hBM-MSCs* show higher level of Jagged1 and Notch1 as compared to hBM-MSCs. In parallel, we observed that hBM-MSCs* failed to inhibit AML proliferation and were more potent than hBM-MSCs in mediating AML chemoresistance. In agreement with previous studies [12, 27], our data show that Notch was expressed by AML samples and MSCs from both normal donors and patients, thus indicating the possibility of crosstalk between the two cell types through this developmental pathway. This hypothesis is clearly supported by the observation that bone marrow leukemia cells expressed higher levels of Notch1 and Notch2 than peripheral blood-derived leukemia cells, thus suggesting that the interaction of leukemia cells with bone marrow stromal cells actively involves the Notch pathway.

Previous studies described the poor activation of Notch in AML samples [11, 12, 28]. Nevertheless, accumulating evidence highlighted the role of Notch in the interaction between bone marrow microenvironment and leukemia cells by showing that hBM-MSCs are capable of inducing activation of Notch signalling in different cancer cells, including multiple myeloma, CLL, and T-ALL [4, 5, 29, 30]. Regardless of FAB classification and genetic abnormalities, we found that 50% of AML cells expressed the activated form of Notch receptors, which were up-regulated following the co-culture with either hBM-MSCs or hBM-MSCs*. Notably, Hes1-negative AML cells were induced to express Hes1 *de novo* following co-culture with hBM-MSCs. Putting these observations together, we hypothesized that Notch could be more expressed and activated in bone marrow, and such activation could be critical to mediate AML chemoresistance. Indeed, the inhibition of hBM-MSC-dependent Notch activation in Jurkat or myeloma cells sensitized the latter to chemotherapy [29, 30]. Accordingly, abrogation of chemoresistance was achieved when pan-Notch inhibitors, such as GSI-IX, GSI-XII, blocking antibody combination or SAHM1, were added to the co-culture. Consequently, we proposed a role for Notch inhibition in preventing hBM-MSCs*-induced chemoresistance of AML cells, and we tried to understand the molecular mechanisms involved. Apoptosis is a cascade of biochemical reactions involving activation of pro-apoptotic proteins of Bcl-2 family leading to activation of caspases, which are effectors of apoptosis. In AML, Bcl-2 inhibition induces apoptosis in a Bak/Bax-dependent manner, thus overcoming intrinsic and microenvironment-induced drug resistance [31]. We show here that Notch inhibition through GSIs substantially enhances Bax/Bcl-2 ratio;in parallel, we found that GSIs were also capable of decreasing the levels of full-length caspase3. These data suggest that the activation of Bax/caspase3 axis could be the consequence of Notch inhibition leading to prevention of hBM-MSCs*-mediated drug resistance. In addition, genetic or pharmacological inhibition of Notch was associated with the decrease of AKT, NF-κB and STAT3 proteins, which all control proliferation and/or cell death, and are targets of caspases [32-34]. In particular, mTor/AKT pathway is constitutively activated in 50-70% of AML cases, and mTor inhibitors, such as Rapamycin and Everolimus [35], have been proved effective, although highly toxic [32], in patients with relapsed/refractory AML. We observed that both pharmacological and genetic inhibition of Notch signalling is associated with decrease of AKT levels, making Notch inhibition an attractive alternative to target AKT/mTor axis. Thus, Notch inhibition could be achieved through many strategies including GSIs, SAHM1 and Notch blocking antibodies.

Blocking antibodies, such as Trastuzumab, Bevacizumab and many others, have shown to be highly effective as anti-cancer therapy [19]. They represent also a useful tool to experimentally investigate the role of specific receptors, by blocking their activation. In order to investigate the contribution of each Notch receptors in the crosstalk between AML cells and hBM-MSCs*, we added specific blocking antibodies to the co-culture. We found that Notch1 and Jagged1 blockade abrogated hBM-MSCs*-mediated AML chemoresistance. Other studies have described the involvement of Notch1 and Jagged1 in microenvironment-mediated chemoresistance: indeed, the inhibition of Notch1 up-regulation in multiple myeloma is sufficient to abrogate hBM-MSC-induced chemoresistance [30]. Similarly, Jagged1 genetically silenced in tumor cells or tumor microenvironment leads to the reduction of AML symptoms and sensitizes tumor cells in ovarian cancer [26, 29]. However, in our co-culture system, the chemoresistance abrogation mediated by the addition of anti-Notch1 and anti-Jagged1 was only partial, thus giving the cue for a possible role of other Notch components in this phenomenon. We have previously shown that Notch receptors involved in stromal cell-mediated chemoresistance were Notch1, Notch2 and Notch4 for CLL, and Notch3 and Notch 4 for B-ALL [4, 5]. Here, we found in AML that the blockage of Notch2 and Jagged2 was also capable of partially restoring the chemosensitivity, while Notch4 and Dll3 blockade abrogated totally AML chemoresistance.

In conclusion, our observation that Notch signalling plays a crucial role in AML cell survival mediated by bone marrow stromal cells may represent the biological basis for new treatment strategies that may become clinically relevant for AML eradication. In fact, the role of Notch pathway has been already described in other malignancies, such as acute lymphoblastic leukemia, and this finding has made a number of Notch pharmacological modulators available for clinical trials. Considering that non-promyelocytic AML is the most frequent acute leukemia in adults, still without definitively curative approaches for most patients lacking bone marrow transplantation chance, the availability of specific inhibitors affecting stromal support to AML cells inside bone marrow could represent an important tool to improve the prognosis of AML patients.

## MATERIALS AND METHODS

### Chemicals and antibodies

The antibodies used for FACS analysis were: mouse IgG2b-FITC, goat IgG-PE, anti-Jagged1-FITC, anti-Dll3-PE (all from R&D System, Minneapolis, MN), mouse IgG2a-PE, mouse IgG1κ-PE, mouse IgG1-Alexa Fluor 488,anti-Notch1-PE, anti-Notch2-PE, anti-Notch3-PE, anti-Notch4-PE, anti-Dll1-PE, anti-Dll4, anti-Bax-Alexa Fluor 488 (all from Biolegend, San Diego, CA) and rabbit anti-Bcl-2-FITC (DAKO). For leukemia cell identification we used anti-CD45-VioBlue, anti-CD45-APC-Vio770, anti-CD34-PerCP and anti-CD117-APC (all from MiltenyiBiotec, Germany). The antibodies employed for western blot analysis anti-Notch2, anti-Notch4 were from Santa Cruz (Biotechnology, Dallas, TX), anti-GAPDH and HRP conjugated secondary antibodies against mouse, rabbit or goat were from Sigma Aldrich. All the other antibodies used for Western blot were from Cell Signalling. Neutralizing antibodies,all used at a final concentration of 5 μg/ml, were: anti-Notch1, anti-Notch3,anti-Jagged1, anti-Jagged2, anti-Dll1 and anti-Dll4 (R&D Systems); anti-Notch-4 (Santa Cruz Biotechnology); anti-Dll3 (CST, Boston, MA). Recombinant human Jagged-1 and Jagged-2 were from R&D System. GSI-IX (DAPT) was purchased from Stemgent (Cambridge, MA) GSI-XII and SAHM1 were from Merck Millipore (Darmstadt, Germany). Cytarabine (Ara-C), Etoposide (Eto) and Idarubicin (Ida) were provided by Pharmacy Unit of the University Hospital of Verona.

### Patients, samples and cell lines

All cell samples were collected from newly diagnosed AML patients and healthy donors after written informed consent, as approved by the Ethical Committee of AziendaOspedalieraUniversitariaIntegrata Verona (N. Prog. 1828, May 12, 2010 - *‘Institution of cell and tissue collection for biomedical research in Onco-Hematology’*). In detail, AML cells were obtained from bone marrow (*n* = 28) or peripheral blood samples (*n* = 16) of patients with AML at diagnosis ( > 90% of leukemic cells) (Table S1A). Human BM-MSCs were obtained from 12 healthy donors and 12 AML patients (Table S1B). They were expanded in complete MEM-α (MEM-α supplemented with 10% fetal bovine serum (FBS), 1% L-Glutamine and 1% penicillin/streptomycin).

Human AML cell lines (HL-60, THP1, U937), CML cell line (K562), and T-ALL cell line (CEM) were cultured in complete RPMI (RPMI supplemented with 10%FBS, 1% L-Glutamine and 1% Penicillin/Streptomycin). HEK-293 were maintained in DMEM supplemented with 10% FBS, 1% L-Glutamine and 1% Penicillin/Streptomycin. Cell lines were validated for STR prior being purchased and were routinely verified to be Mycoplasm-free.

### Cell cultures and co-cultures

BM-MSCs were expanded and characterized as previously described [4, 5]. Co-culture experiments were performed in complete RPMI on a confluent monolayer of hBM-MSCs or hBM-MSCs* in 96-well plates: 10^5^ AML cells or 2 × 10^4^cells from AML cell lines were seeded alone or on stromal monolayer supplemented with Notch modulators or different drugs used in AML treatment, including Cytarabine (Ara-C), Etoposide (Eto), Idarubicin (Ida). To determine EC50 dose for each drug and modulator, we performed the colorimetric 3-[4,5-dimethylthiazol-2-yl]-2,5-diphenyltetrazolium bromide (MTT, Sigma-Aldrich) metabolic activity assay. Apoptosis was evaluated by AnnexinV-FITC assay (MiltenyiBiotec). To study cell proliferation, AML cells were labelled with carboxyfluoresceinsuccinimidylester (CFSE) (Life Technologies) (5mM). For primary leukemia cells proliferation assay, complete RPMI medium was supplemented with IL-3, IL-6 and SCF, as previously described (15). After 4 days, AML cells were harvested, stained with anti-CD45 PerCP-Vio700 antibody and analyzed by FACS CantoII (BD Biosciences). MTT, AnnexinV-FITC and CFSE assay are described in detailed in Supplementary Methods.

### Gene reporter assay

THP1 cells were transfected with reporter plasmids encoding for an inducible RBP-Jk-responsive GFP reporter (Qiagen) using MACSfectin transfection reagent (MiltenyiBiotec). GFP signal were quantitatively measured by flow cytometry. Notch activity was determined by normalizing the activity of RBP-Jk-GFP to that of CMV-GFP plasmid.

### Generation of RBP-jk knockdown cell lines

The pLKO.1-puro-CMV-TurboGFP lentivirus plasmid (SHC003) was obtained from Sigma. For gene silencing, shRNA plasmids targeted to RBP-jk, TRCN0000016207 (shRBP-jk), Scramble shRNA SHC016 were purchased from Sigma, as well asLentiviral Packaging Mix (SHP001). Plasmids were co-transfected into 293T cells usingMACSfectin transfection reagent (MiltenyiBiotec), at a ratio of 1:1(plasmid of shRNA:Packaging Mix). Media were replaced with DMEM + 10% FBS at 16 hours after transfection; viruses were collected at 48 and 72 hours after transfection and filtered through a 0.45 μm filter. HL-60 cells and THP1 cells were transduced by the lentivirus in the presence of the polycation Polybrene, and the stably transduced cells were then selected by puromycin (1μg/ml) for 4 weeks.

### Adipogenic and osteogenic differentiation

hBM-MSCs or hBM-MSCs* were seeded in 24 wells plates. Once reached the confluence, cells were induced to differentiate by replacing growth medium by adipogenic or osteogenic differentiation media. Adipogenic differentiation medium consisted in DMEM supplemented with 18% FBS, 10mg/ml IBMX (Sigma-Aldrich), 100U/ml Insulin (Lilly) and 1mM Dexamethasone (Sigma-Aldrich). For Osteogenic differentiation, cells were incubated with StemMACSOsteo Diff Medium (MiltenyiBiotec). Differentiation media were changed each 3 days and cells were allowed to differentiate for 3 weeks; then cells were characterized with Oil Red O staining and Alizarin red for adipogenic andosteogenic differentiation, respectively.

### Western blotting

Cells were lysed with an appropriate amount of RIPA buffer (25 nMTris pH 7.6, 150mM NaCl, 1% NP40, 1% Na-deoxycholate, 0.1% SDS) supplemented with complete Protease Inhibitor (Roche) and 1 mM Na_3_VO_4_. Proteins were quantified using BCA protein assay kit (Thermo Scientific, Waltham, MA) and separated on a 10% or 12% polyacrylamide gel. Subsequently, proteins were transferred onto nitrocellulose membrane (GE Healthcare), labelled with appropriate antibody and acquired by LAS4000 (GE Healthcare) instrument. Densitometric analyses were performed on scanned immunoblot images using the ImageJ analysis tool (National Institutes of Health, Bethesda, Maryland). GAPDH density was used to normalize density of each protein.

### Statistical analysis

Statistical analysis was performed using GraphPad Prism software (La Jolla, CA). Data were expressed as mean ± standard error means (SEM). Student *t*-test was used to compare 2 groups and one-way ANOVA followed by the Tukey's range test were applied to compare multiple groups. Mann-Whittney test was used for non-coupled, non-parametric comparison.

### SUPPLEMENTARY MATERIAL FIGURES AND TABLES


