# Supplementary material for: Notch signalling drives bone marrow stromal cell-mediated chemoresistance in acute myeloid leukemia
Source: Oncotarget. 2016 Mar 7;7(16):21713–27. doi: 10.18632/oncotarget.7964 (PMC5008317; doi:10.18632/oncotarget.7964)
Supplement: Supplementary file 1 [file oncotarget-07-21713-s001.pdf]

Notch signalling drives bone marrow stromal cell-mediated chemoresistance in acute myeloid leukemia

Supplementary Material

Chemicals and antibodies

The antibodies used for FACS analysis were: mouse IgG2b-FITC, goat IgG-PE, anti-Jagged1-FITC, anti-Dll3-PE (all from R&D System, Minneapolis, MN), mouse IgG2a-PE, mouse IgG1κ-PE, mouse IgG1-Alexa Fluor 488,anti-Notch1-PE, anti-Notch2-PE, anti-Notch3-PE, anti-Notch4-PE, anti-Dll1-PE, anti-Dll4, anti-Bax-Alexa Fluor 488 (all from Biolegend, San Diego, CA) and rabbit anti-Bcl-2-FITC (DAKO).The antibodies employed for western blot analysis anti-Notch2, anti-Notch4 were from Santa Cruz (Biotechnology, Dallas, TX),anti-GAPDH and HRP conjugated secondary antibodies against mouse, rabbit or goat were from Sigma Aldrich. All the other antibodies used for western blot were from Cell Signaling. Neutralizing antibodies, all used at a final concentration of 5 μg/ml, were: anti-Notch1, anti-Notch3, anti-Jagged1, anti-Jagged2, anti-Dll1 and anti-Dll4 (R&D Systems); anti-Notch-4 (Santa Cruz Biotechnology); anti-Dll3 (CST, Boston, MA). Recombinant human Jagged-1 and Jagged-2 were from R&D System. GSI-IX (DAPT) was purchased from Stemgent (Cambridge, MA) GSI-XII and SAHM1 were from Merck Millipore (Darmstadt, Germany). Cytarabine (Ara-C), Etoposide (Eto) and Idarubicin (Ida) were provided by Hospital Pharmacy of the University Hospital of Verona.

Transwell co-culture experiments

Co-culture experiments without cell contact were performed in 24-wells plates. 10^5 AML-cell line cells or 10^6 AML primary blast cells were grown in a membrane Transwell insert (Corning) placed in a well containing a confluent monolayer of hBM-MSCs or hBM-MSCs* in complete RPMI.

MTT viability assay

To study the specific relative basal sensitivity of AML cells to modulators of pathway as well as to different drugs, AML cells were cultured for 48 hours in presence of increasing concentrations of each modulator or drug. To determine EC50 dose for each drug and modulator, we performed the colorimetric3-[4,5-dimethylthiazol-2-yl]-2,5-diphenyl tetra-

zolium bromide (MTT, Sigma-Aldrich) metabolic activity assay. AML lines (10^4 cells per wells) or AML primary cells (10^5 cells per wells) were seeded in 96 wells plates, with increasing concentrations of different drugs. Cell viability was then assessed by adding 10 µL of MTT into each well and keeping in incubator for 3 hours. Metabolically active, viable cells converted MTT into a colored formazan, which was solubilized with a volume of acid isopropanol equal to the volume of cell suspension. The product was then measured at 570nm in a spectrophotometric microplate reader (PerkinElmer VICTORX4). The viability was expressed as the percentage of optical density of treated cells compared to optical density of cells treated with the specific vehicle. The effective concentration to induce 50% reduction of AML cells viability (EC50) derived from the equations that best fit the linear range of the dose-response curve. Each experimental condition was done in hexaplicate and repeated at least twice.

Apoptosis, cell proliferation and TOPRO-3 viability assay

Apoptotic rate of AML cells was assessed using FITC-conjugated AnnexinV (AnnexinV-FITC) and Propidium Iodide (PI) staining. AML cells were washed twice with PBS and then stained with APC-conjugated anti-CD45 for 10 minutes in the dark at room temperature. Cells were resuspended in binding buffer (MiltenyiBiotec), and AnnexinV-FITC (MiltenyiBiotec) was added at 1 µg/mL final concentration. The mixture was incubated at room temperature for 15 minutes in dark. Membrane integrity was assessed by PI staining immediately before flow cytometry analysis on a FACS Canto II.

Cell proliferation was evaluated by flow cytometry analysis after carboxyfluoresceinsuccinimidylester(CFSE) (Life Technologies) staining. Briefly, human AML cells were washed twice with PBS and resuspended in 0.1% PBS-BSA (Bovine Serum Albumin), stained with CFSE (5mM) for 10 minutes in the dark at 37°C and incubated 5 minutes on ice. Stained cells were used in different culture and co-culture experiments. At the end of the culture or co-culture, AML cells were pelleted by centrifugation and washed three times with fresh media. Then, AML cells were harvested and stained with anti-CD45-PerCP. In order to discriminate live cell population, samples were stained with TO-PRO-3 (1µM) and analyzed by FACS. Relative cell proliferation were expressed as the percentage of CFSE median fluorescence of treated cells compared to that of cells treated with the specific vehicle.

MayGrunwald-Giemsa staining

We applied this method to study whether the treatments with GSIs and Idarubicin affected hBM-MSCs* morphology. Briefly, adherent hBM-MSCs* were washed with PBS to remove AML cells, then cells were stained with three decreasing concentrations of MayGrunwald-Giemsa solution, i.e. 3 minutes with pure 100% MayGrunwald, 2 minutes with 1/3 diluted MayGrunwald solution, and finally 15 minutes with 1/10 diluted Giemsa solution. Cells were then washed with distillated water and dried at room temperature. Stained cells were acquired with Axiovert Z1 Observer Microscope (Zeiss).

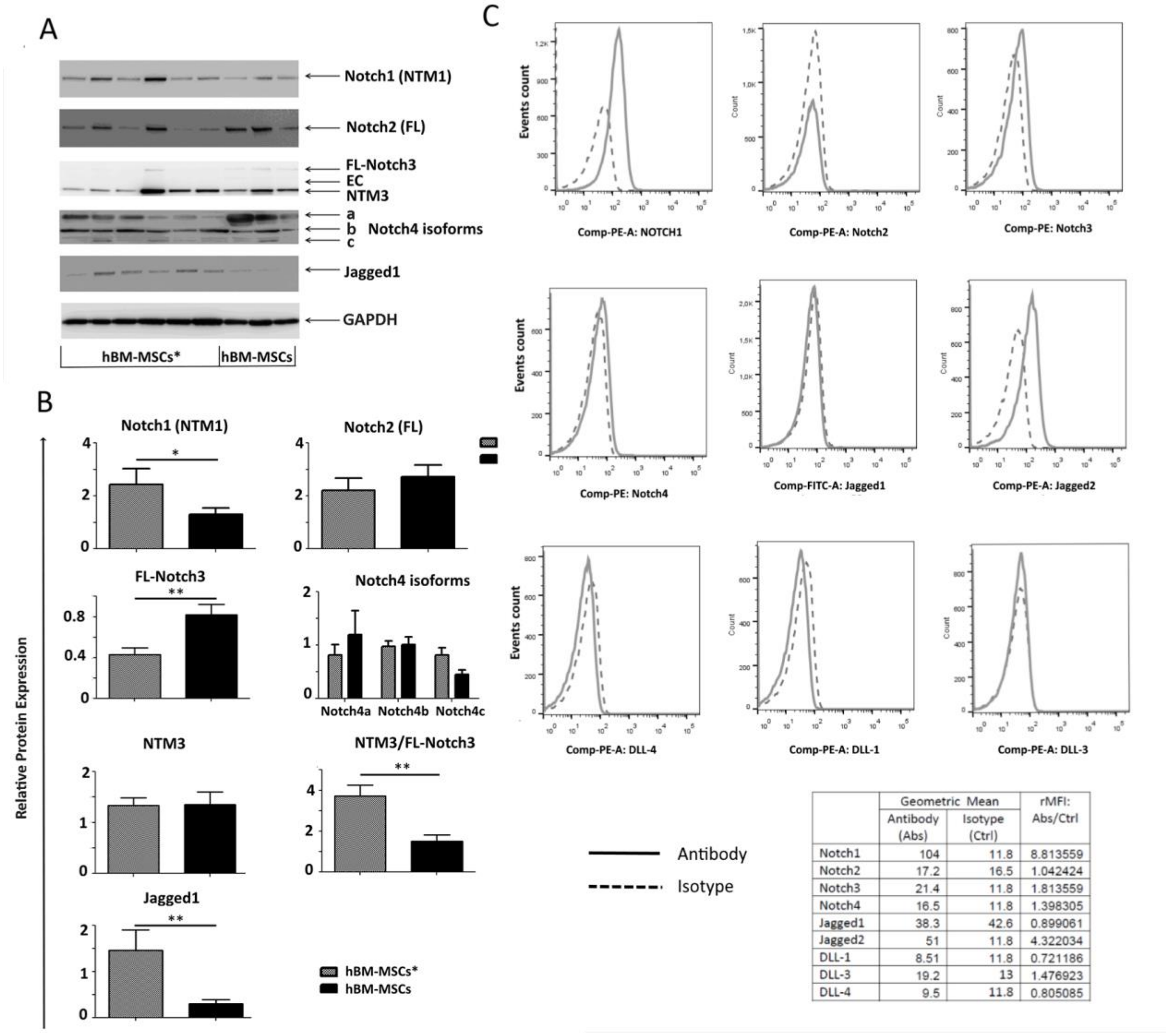

	Geometric Mean		rMFI: Abs/Ctrl
	Antibody (Abs)	Isotype (Ctrl)	
Notch1	104	11.8	8.813559
Notch2	17.2	16.5	1.042424
Notch3	21.4	11.8	1.813559
Notch4	16.5	11.8	1.398305
Jagged1	38.3	42.6	0.899061
Jagged2	51	11.8	4.322034
DLL-1	8.51	11.8	0.721186
DLL-3	19.2	13	1.476923
DLL-4	9.5	11.8	0.805085

Figure S1. Analysis of Notch expression in hBM-MSCs and AML samples

A) Representative western blot analysis of the expression of Notch components in hBM-MSCs* and hBM-MSCs. Images are representative of at least 3 independent experiments performed on 12 hBM-MSCs* and 12hBM-MSC samples. B) Relative quantification of protein expression as compared to GAPDH. Data are expressed as the mean ± SEM of at least 3 independent experiments: *p<0.05, **p<0.01. HEK-293 cell line was used as positive control. B) Example of flow cytometry plot to evaluate expression level of Notch components in AML samples. Flow cytometry analysis was performed with fluoruophore-coupled antibodies and their specific isotype counterpart. Data are expressed as the ratio of the geometric mean of antibody fluorescence and isotype control fluorescence.

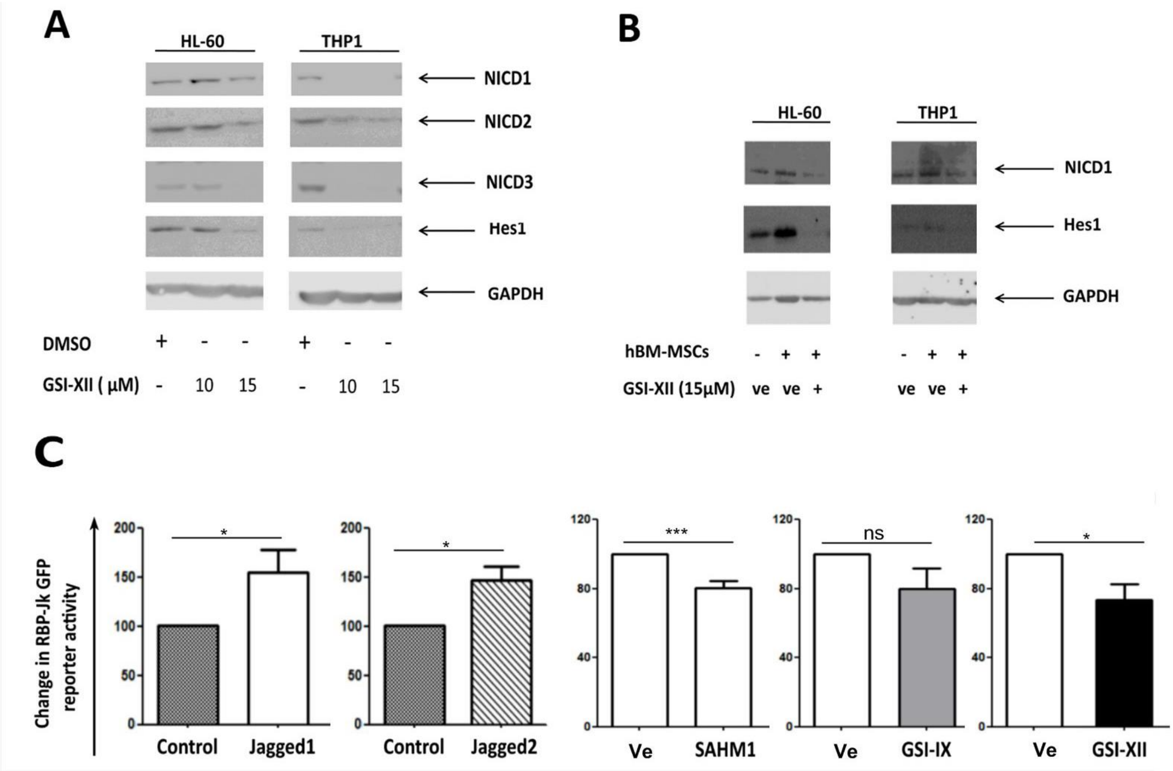

Figure S2. Notch activation status in AML cells after GSI treatment in presence or absence of hBM-MSC.

A) AML cell lines in culture, B) or in co-culture with hBM-MSCs were treated with GSI-XII before protein collection. Images are representative of 3 independent experiments. C) THP1 cells were transfected with RBP-Jk GFP reporter gene and treated with Notch modulators 2 hours before transfection, i.e. a combination of anti-Notch1, 2, 3 and 4 blocking antibodies (pan-R-abs, pan Receptor blocking antibodies), each antibody at a final concentration of 5µg/ml, SAHM1 (20µM) or GSI-IX (15µM) or GSI-XII (10µM), which were maintained in the medium during and after transfection. GFP levels were analyzed through flow cytometry after 72 hours of co-culture. Data were normalized in percentage with THP1 cells transfected with CMV-GFP plasmid and are expressed as mean ± SEM of 2 independent experiments: *p<0.05, ***p<0.001, NS: not significant. “ve”: vehicle=DMSO.

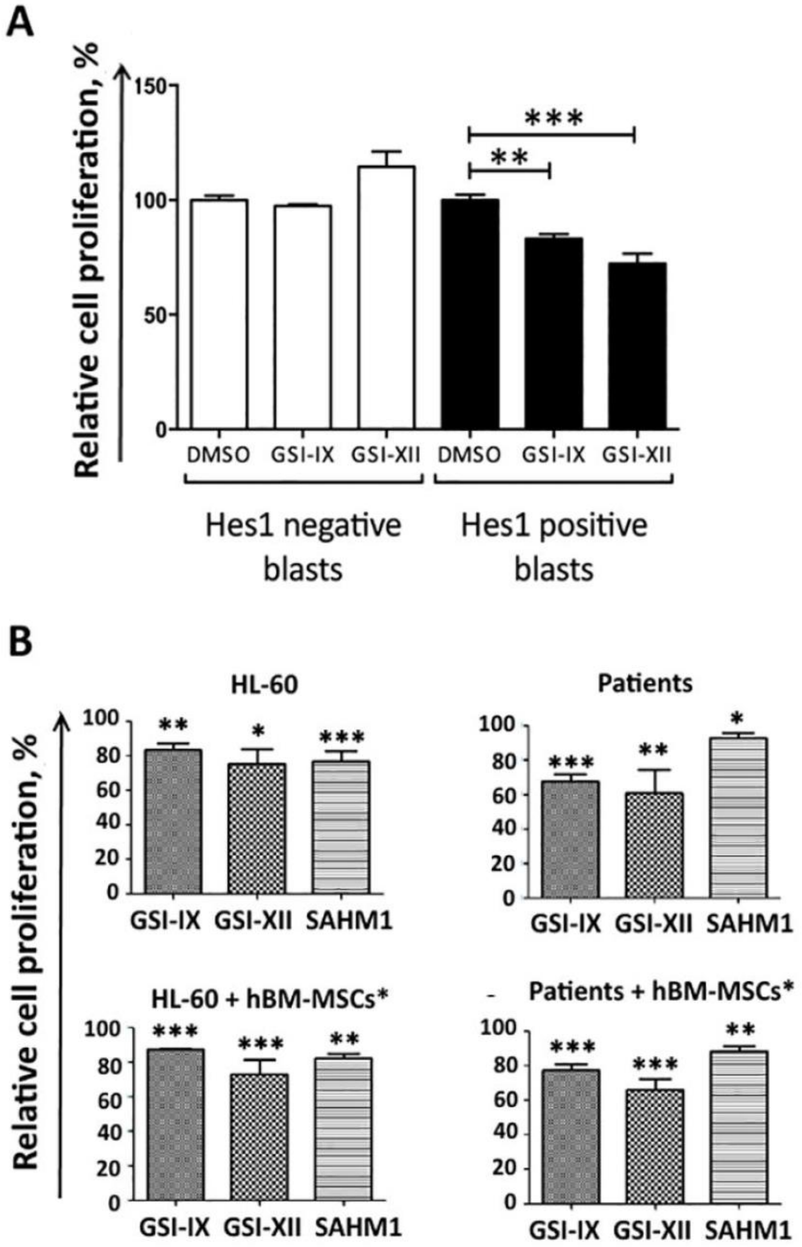

Figure S3. Notch signalling controls AML cell proliferation in culture and in co-culture conditions.

A) Effects of 48 hour-treatment with GSIs on Hes1-negative and Hes1-positive leukemia cells. B) CFSE-stained HL-60 or primary AML cells were treated with either GSI-IX (15µM), GSI-XII (10µM) or SAHM1 (20µM), and cultured alone or in presence of hBM-MSCs*. After 4 days, cells were collected and analyzed by FACS. Data are normalized to the proliferation of HL-60 and primary leukemia cells alone (upper panels) or HL-60 and primary leukemia cells co-cultured with hBM-MSCs* (lower panels). Data are expressed as the mean ± SEM of 3 independent experiments: *p<0.05, **p<0.01, ***p<0.001.

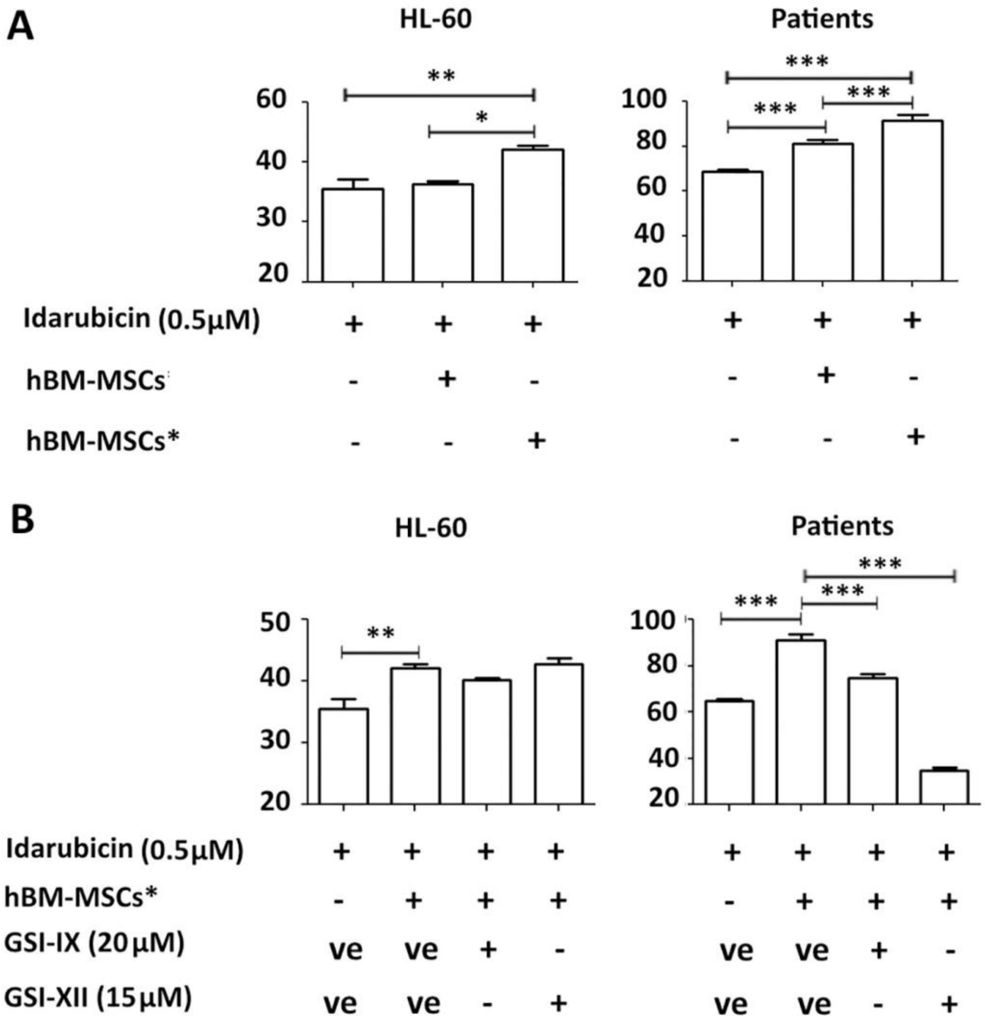

Figure S4. hBM-MSCs* control AML cells chemoresistance in Transwell® co-culture.

A) AML cells were cultured in a Transwell® insert (4µm) in presence or in absence of hBM-MSCs or hBM-MSCs* in presence of Idarubicin (0.5 µM). B) AML cells were cultured in a Transwell® insert with hBM-MSCs* in presence of Idarubicin (0.5 µM), with or without GSI-IX (20µM) or GSI-XII (15µM). After 48 hours of treatment, AML cells were harvested and seeded in 96 well plates. Then cell viability was analyzed by MTT test. Data are represented as mean ± SEM of 2 independent experiments performed in octuplicate. One way Anova was used for statistical analysis: * $p<0.05$, ** $p<0.01$, *** $p<0.001$. ve=DMSO.

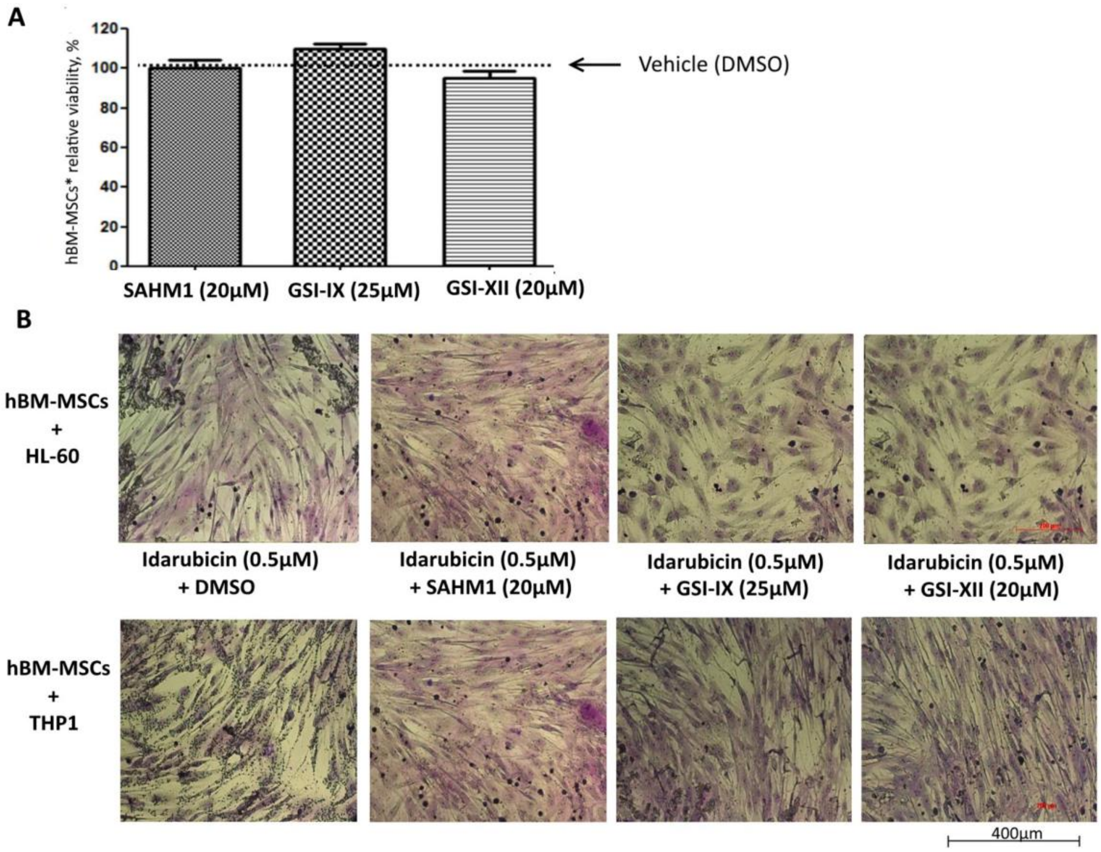

Figure S5. Viability and morphology of hBM-MSCs and hBM-MSCs* after treatment with GSI-IX or GSI-XII. A) hBM-MSCs* alone were treated for 48 hours with SAHM1(20µM) or GSI-IX (25µM) or GSI-XII (20µM); MTT assay was performed and relative viability was determined by normalizing data to DMSO treated cells. B) hBM-MSCs were co-cultured with AML cells in presence of Idarubicin (0.5µM) alone or with either SAHM1 (20µM) or GSI-IX (25µM) or GSI-XII (20µM); after 48 hours AML cells were removed by PBS washing, and adherent hBM-MSCs were stained with May Grunwald-Giemsa and observed under a light microscope Axiovert Z1 (original magnification ×20).

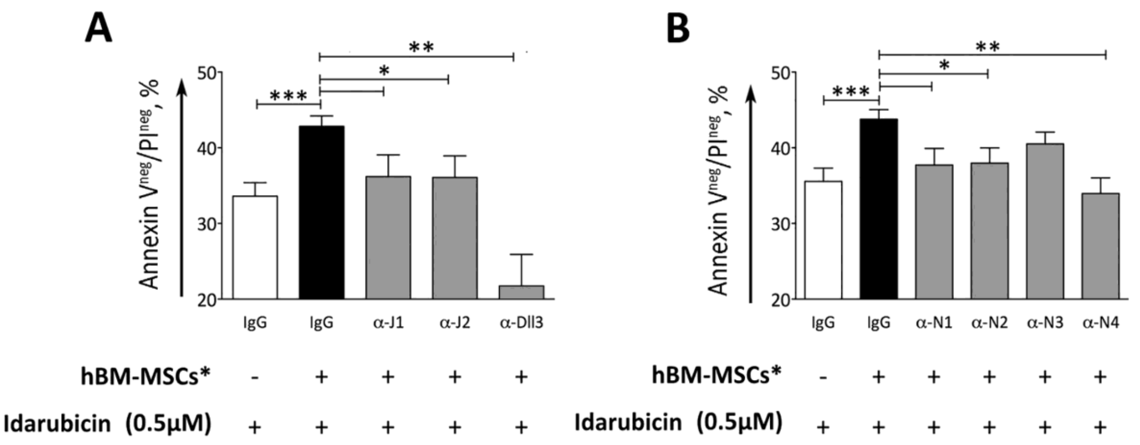

Figure S6. Notch blocking antibodies abrogate hBM-MSC*-induced chemoresistance.

AML cells were cultured alone or co-cultured with hBM-MSCs* in presence of Idarubicin (0.5μM). A) Addition of Notch ligand blocking antibody to the co-cultures α-J1 (anti-Jagged1), α-J2 (anti-Jagged2), α-Dll3 (anti-Dll3), B) or Notch receptors blocking antibodies: α-N1 (anti-Notch1), α-N2 (anti-Notch2), α-N3 (anti-Notch3), α-N4 (anti-Notch4). All blocking antibodies were used at a final concentration of 5μg/ml. Cell viability was analyzed through AnnexinV/PI assay. Data are expressed as mean ± SEM of 3 independent experiments: *p<0.05, **p<0.01, ***p<0.001.

Table S1. Characteristics of AML patients.

A) AML blast cells were obtained after informed consent from bone marrow or peripheral blood samples of 44 patients with AML at diagnosis. B) 12 bone marrow samples were employed to obtain hBM-MSCs*. NA: not available, this group includes both patients without diagnosed genetic abnormalities as well as patients whose analyses are not available

A

Samples	Sex	Median Age	FAB	Molecular Features
44<br>(28 from bone marrow,<br>16 from peripheral blood)	F = 16<br>M = 28	51 [19-78]	MO = 6<br>M1 = 6<br>M2 = 11<br>M3 = 4<br>M4 = 5<br>M4Eo = 3<br>M5 = 5<br>NA = 4	AML1-ETO = 3<br>PML-RARA = 4<br>NPM1 = 4<br>FLT3-ITD = 4<br>MLL =2<br>CBFB-MYH1 = 2<br>FLT3 = 2<br>NA = 23

B

Samples	Sex	Median Age	FAB	Molecular Features
12<br>(all from bone marrow)	F = 5<br>M = 7	54 [27-78]	MO = 2<br>M1 = 2<br>M2 = 2<br>M3 = 1<br>M4 = 1<br>M4Eo = 1<br>NA = 3	AML1-ETO = 1<br>NPM1 = 1<br>CBFB-MYH1 = 1<br>FLT3 = 1<br>PML-RARA = 1<br>NA = 7

Table S2. Notch status in AML samples used in functional assays

A) Before functional assays, Notch activation status was analyzed in AML samples through western blot. Samples showed Hes1 expression and an active/cleaved-Notch receptor were considered to display activation of Notch signalling. NA: not available..

Patients	FAB	Blast count	Figures or tables	Notch activation status
Patient2	M1	80%<	2,3, 4, 6 , S3 S4, tables S3, S4	+
Patient3	NA	80%<	2, 4, 5,6, 7, S3,S4, table, S3,S4, S5	+
Patient4	M0	80%<	2, 4, 6,7, S3, S4, tables, S3,S4.	+
Patient5	M2	80%<	2, 5,7,	+
Patient6	M0-M1	80%<	2, 5,6, S3, S4	-
Patient7	M0	80%<	2, 5,6	+
Patient11	M5	80%<	2, 4, 5,6, S4, tables, S3,S4.	+
Patient12	M5	80%<	2, 4, 5,6	+
Patient13	NA	80%<	2, 3,7	+
Patient14	NA	80%<	2, 3,7	+
Patient8	M2	80%<	2, 3, 4,5	+
Patient9	M0	80%<	2,5,	+
Patient10	M2	80%<	2, 3, 4,5, S3	-
Patient15	M1	80%<	2, 3,7	+
Patient16	M2	80%<	2, 5, S3	-
Patient17	M2	80%<	2, 3, 4, 5, 6,7, S4, table, S3,S4.	+
Patient18	M2	80%<	2, 3, 7	+
Patient19	NA	80%<	2,3	+
Patient20	NA	80%<	2, 4,5,6, S4, S3,S4, table, S3,S4.	+

Table S3. AML cell viability upon treatment with different Notch modulators.

AML cells were treated with different modulators in culture or co-culture with hBM-MSCs* for 4 days. TOPRO-3 negative cells were quantified by FACS analysis and reported as mean ± SEM of 3 independent experiments involving 5-12 patients.

rh-Jagged : Recombinant human Jagged

	relative AML cell viability (%)					
	**rh-Jagged1**	**rh-Jagged2**	**DMSO**	**GSI-IX**	**GSI-XII**	**SAHM1**
**Patients**	106.8 ± 1.055	103.8± 1.3.8	102.8 ± 3.209	85.28 ± 5.406	45.48 ± 16.82	120.0 ± 20.06
**Patients + hBM-MSCs***			98.29 ± 12.84	99.71 ± 2.254	86.29 ± 5.537	95.81 ± 15.60
**HL-60**	102.7 ± 1.036	102.8 ± 0.968	90.12 ± 8.477	101.0 ± 1.338	74.98 ± 9.120	101.2 ± 2.088
**HL-60 + hBM-MSCs***			93.32 ± 3.231	97.17 ± 0.3333	92.71 ± 1.833	78.50 ± 9.582
**THP1**	93,80 ± 2,612	94.04 ± 2.797	94.13 ± 2.140	95.28 ± 1.434	93.04 ± 3.264	98.25 ± 0.250
**THP1 + hBM-MSCs***			88.76 ± 2.250	95.93 ± 0.6438	95.66 ± 0.7755	79.18 ± 2.074

Table S4. GSI-XII abrogates hBM-MSC-mediated chemoresistance.

Viability of AML cells in culture or co-culture with hBM-MSCs in presence of either Ara-C (10µM), Etoposide (10µM) or Idarubicin (0.5µM) and increasing concentrations of GSI-XII. After 48 hours, cells were collected and analyzed by AnnexinV/PI assay. Data are expressed as mean ± SEM of 3 independent experiments. Statistical analysis was performed with one-way Anova comparing all the culture conditions with AML + hBM-MSCs in presence of DMSO. *p<0.05, **p<0.01, ***p<0.001.

Cell viability (%)	AML alone	AML + hBM-MSCs				
	DMSO	DMSO	GSI-XII 5µM	GSI-XII 10µM	GSI-XII 15µM	GSI-XII 20µM
				Ara-C		
**Patients**	15.89 ± 3.455 *p	30.29 ± 4.706	29.71 ± 4.267 ns	26.16 ± 3.656 ns	24.85 ± 4.062 ns	21.38 ± 3.150 *p
**HL-60**	33.04 ± 4.431 **p	49.69 ± 1.463	43.05 ± 3.369 ns	37.65 ± 2.997 *p	22.34 ± 2.835 ***p	20.88 ± 2.068 ***p
**THP1**	15.15 ± 2.540 ***p	32.32 ± 1.630	35.23 ± 1.498 ns	30.42 ± 2.428 ns	24.50 ± 1.444 **p	23.10 ± 1.379 **p
**U937**	18.03 ± 2.535 **p	35.50 ± 3.402	42.70 ± 1.900 ns	34.78 ± 3.157 ns	20.84 ± 1.921 **p	20.88 ± 2.662 ***p
				ETOPOSIDE		
**Patients**	29.09 ± 4.166 ***p	52.43 ± 2.773	51.62 ± 1.838 ns	42.77 ± 1.747 *p	36.50 ± 2.890 **p	30.10 ± 2.602 ***p
**HL-60**	11.29 ± 1.704 *p	20.35 ± 5.178	9.305 ± 1.255 **p	7.514 ± 0.7850 ***p	9.370 ± 0.5800 *p	5.933 ± 0.3858 ***p
**THP1**	4.948 ± 1.014 ***p	22.70 ± 1.809	20.52 ± 1.610 *p	18.23 ± 2.045 *p	13.85 ± 2.789 *p	13.28 ± 1.355 **p
**U937**	9.737 ± 4.079 *p	25.84 ± 5.888	17.26 ± 0.9352 ns	8.008 ± 1.385 *p	6.096 ± 1.227 *p	5.968 ± 1.598 *p
				IDARUBICIN		
**Patients**	26.26 ± 2.072 ***p	50.02 ± 2.519	44.07 ± 2.277 ns	33.17 ± 1.356 ***p	27.14 ± 1.508 ***p	21.18 ± 2.780 ***p
**HL-60**	11.36 ± 1.195 ***p	26.41 ± 3.332	16.18 ± 0.8542 *p	14.04 ± 0.6836 **p	11.79 ± 1.208 **p	10.25 ± 1.169 ***p
**THP1**	12.68 ± 1.224 ***p	23.32 ± 1.214	16.47 ± 1.191 **p	18.52 ± 2.888 ns	16.51 ± 0.6592 **p	15.35 ± 0.5377 **p
**U937**	3.683 ± 0.7641 **p	14.49 ± 3.247	17.93 ± 2.058 ns	11.30 ± 0.4933 *p	5.750 ± 0.7500 **p	1.130 ± 0.3800 **p
